# Litter-Induced Reduction in Ecosystem Multifunctionality Is Mediated by Plant Diversity and Cover in an Alpine Meadow

**DOI:** 10.3389/fpls.2021.773804

**Published:** 2021-11-25

**Authors:** Zhouwen Ma, Jing Wu, Lan Li, Qingping Zhou, Fujiang Hou

**Affiliations:** ^1^State Key Laboratory of Grassland Agro-Ecosystems, Key Laboratory of Grassland Livestock Industry Innovation, Ministry of Agriculture and Rural Affairs, College of Pastoral Agriculture Science and Technology, Lanzhou University, Lanzhou, China; ^2^Institute of Qinghai-Tibet Plateau, Southwest Minzu University, Chengdu, China

**Keywords:** alpine meadow, ecosystem multifunctionality, litter, plant diversity, plant functional composition

## Abstract

Litter has been shown to alter the structure and functions of grassland ecosystems, and a knowledge of the effects of litter is essential for understanding the dynamics of ecosystem multifunctionality. However, relatively little is known about the effects of plant litter on ecosystem multifunctionality in alpine meadows. A three-year field experiment was conducted to explore how litter manipulation affects ecosystem multifunctionality. The plant litter treatments that were applied consisted of a range of litter mass levels and three dominant plant species, in an alpine meadow on the Qinghai-Tibet Plateau. The results showed that litter mass manipulation had a negative effect on ecosystem multifunctionality and most individual ecosystem functions (species richness, plant cover, and above-ground biomass) but had a positive effect on plant functional group evenness. In particular, the study found that low or medium amounts of litter (≤200gm^−2^) were beneficial in maintaining a high level of ecosystem multifunctionality. Furthermore, a structural equation model revealed that ecosystem multifunctionality was driven by indirect effects of litter mass manipulation on plant functional group evenness, plant cover, and species richness. These results suggest that litter-induced effects may be a major factor in determining grassland ecosystem multifunctionality, and they indicate the potential importance of grassland management strategies that regulate the dynamics of litter accumulation.

## Introduction

Ecosystem multifunctionality is an important manifestation of the value of the ecosystems. It indicates the ability of an ecosystem to simultaneously maintain both above- and below-ground multiple ecological functions or services (Hector and Bagchi, 2007; Zavaleta et al., 2010) and critical for accurately evaluating multi-dimensional spatial pattern and implementing sustainable management (Song et al., 2020a). An understanding of how ecosystem multifunctionality is related to biotic attributes of ecological communities as the focus of attention (Manning et al., 2018). Biodiversity and optimal population allocation are key determinants of ecosystem multifunctionality in natural grassland (Liu et al., 2021) and are driven in particular by plant community functional characteristics (Zhang et al., 2021) and soil microbial communities (Jing et al., 2015). Therefore, further research is needed to explore the key contributions of different biotic factors (e.g., plant litter) in driving ecosystem functioning, which in turn will enable improved grassland management strategies and maintain high levels of ecosystem multifunctionality.

Previous studies have shown that the ecological effects of ecosystem multifunctionality are mainly linked to biodiversity factors (Lefcheck et al., 2015). In particular, species richness and evenness are strongly and positively related to ecosystem multifunctionality (Li et al., 2017). In addition, plant cover has a critical role in sustaining ecosystem multifunctionality (Soliveres et al., 2014). Plant functional composition plays an important part in vegetation dynamics. Shifts in plant functional composition can determine the plant diversity, and this in turn affects ecosystem multifunctionality (Maestre et al., 2012a). Therefore, there is a need to examine the effect of such ecological functions on the ecosystem multifunctionality of alpine meadows with plant litter feedback.

In the diverse ecosystem of grassland, plant litter dynamics are considered to be one of the main factors that can influence ecosystem functions. Multiple plant litter feedbacks occur in terrestrial ecosystems, and they contribute to community functioning (Mariotte et al., 2016; Che et al., 2018). The complex interactions of litter with plant–soil communities are well documented (Eppinga et al., 2011; Veen et al., 2019). Plant litter may promote plant strategies that favor resource allocation for particular modules and thereby drive dynamics that optimize plant functional composition (Kaproth et al., 2013). Litter accumulation influences plant functional composition and diversity directly or indirectly by altering soil microclimate and species recruitment in terrestrial ecosystems (Fang et al., 2012; Yuan et al., 2016). The effects of litter quantity or allelopathic compounds that may favor vegetative growth are also widely recognized mechanisms that lead to altered above- and below-ground ecological functions (Elgersma et al., 2012; Mariotte et al., 2016). Therefore, plant litter may strongly influence ecosystem multifunctionality through physical or chemical drivers.

Meta-analytical studies have shown that a litter mass of less than 200gm^−2^ has a more positive effect on the ecological functioning of plant community (e.g., plant establishment, species richness, and above-ground biomass; Xiong and Nilsson, 1999), and increased seedling survival has been reported where litter accumulation is low (<250gm^−2^) in grassland ecosystems (Loydi et al., 2013). These responses were dependent on the type of litter, with forbs litter having a stronger chemical effect on vegetation than grass litter (Xiong and Nilsson, 1999). It has been reported that the physical barrier created by litter can both reduce light availability and alter the temperature in the soil environment, thereby influencing the composition and productivity of plant communities (Weltzin et al., 2005). The resulting variation would amplify the effects of plant litter on ecosystem multifunctionality. However, to date, little evidence has been found for a relationship between plant litter and ecosystem multifunctionality in alpine meadows, and the role of litter feedback pathways as drivers of ecosystem multifunctionality is unclear.

The Qinghai-Tibet Plateau (QTP), often referred to as the “Roof of the World,” is the largest and highest plateau (altitude>4,000m) in the world, with the most extensive biodiversity history (Myers et al., 2000). Alpine meadows represent the largest ecosystem (accounting for more than 60% of the eco-region of the QTP) and provide a number of important functions and services (Chen et al., 2013). Intensive livestock grazing is one of the main causes of grassland degradation, which in turn affects ecosystem functions (Dong et al., 2013). Although the vegetation on the QTP is generally dominated by several graminoid species, an increasing number of noxious forbs have been rapidly spreading in many areas of the plateau (Tang et al., 2015). Grazing exclusion and moderate grazing have been proposed and subsequently implemented in order to mitigate the degradation of ecosystem functions in alpine meadows, which would be likely to alter both the botanical composition and the productivity of this ecosystem. This would lead to heterogeneity of plant litter accumulation, including an increase in litter mass and changes in litter species composition (Hu et al., 2016; Zou et al., 2016), which would in turn have an impact on litter feedback mechanisms. Therefore, an understanding of such mechanisms is important for elucidating the ecosystem multifunctionality of ecological communities.

In this paper, we describe a three-year litter manipulation experiment designed to investigate three perennial grassland species, consisting of two graminoids (*Elymus nutans* and *Kobresia setchwanensis*) and one noxious forb (*Ligularia virgaurea*), and five litter mass levels, in an alpine meadow grassland on the QTP. These three species are all widely used indicator species in alpine meadows and represent different plant functional groups. The study attempted to explore the regulatory effects of litter manipulation on ecosystem multifunctionality, as the findings would have important implications for ecological conservation and sustainable management of grassland. Previous studies have shown that impacts of litter manipulation can decrease the species richness and species evenness (Lamb, 2008), and promote the transformation in plant functional composition (Amatangelo et al., 2008). These factors are important determinants of ecosystem multifunctionality (Maestre et al., 2012a). Therefore, we hypothesized that litter mass, litter species, and their interaction can significantly alter plant functional composition, species richness, plant functional group evenness, and plant cover, and further influence ecosystem multifunctionality. Based on previous studies on the effects of litter on plant community ecosystem functions, we predicted the net effect of litter addition would result a reduction of ecosystem multifunctionality and that the effect of *L. virgaurea* litter treatment would be stronger. Finally, we constructed the piecewise structural equation models to determine the relative importance and pathways of various drivers regulating the variations in ecosystem multifunctionality. The aims of the study were as follows: (1) to investigate the effects of litter manipulation on ecosystem multifunctionality; (2) to identify the mechanism that drives those effects; and (3) to determine the response of the key mechanisms of ecosystem multifunctionality to litter manipulation.

## Materials and Methods

### Study Site

The experiment site was conducted at an alpine meadow, located in Hongyuan county, Sichuan province, China (31°47' N, 102°33' E, 3,500ma.s.l.) at the eastern Tibetan Plateau. This region climate is continental monsoon with a mean annual temperature of 1.5°C and annual precipitation 747mm (1961–2013) with approximately 80% concentrated in growing season (May–September). The vegetation in alpine meadow dominated by graminoids, such as *E. nutans*, *Carex thibetica*, and *K. setchwanensis*, and perennial forbs, such as *Anemone rivularis*, *Anemone trullifolia*, *Saussurea hieracioides*, *L. virgaurea*, and legumes, is rare. The soil type in this study site is Mat Cry-gelic Cambisol.

### Experimental Design

To determine the effects of the litter of dominant plant species on ecosystem multifunctionality, we conducted a field experiment using a randomized block design in an enclosed area in 2017. Treatments consisted of a range of litter mass levels (0, 100, 200, 400, and 600gm^−2^) for three individual plant species (*E. nutans*, *K. setchwanensis*, and *L. virgaurea*). Each treatment was applied to four 2×2m plots, which were divided randomly into blocks. The individual plots were separated by a buffer zone of >1m. Plant litter was collected from the vicinity of the study site and then air-dried. All standing above-ground dead plant material was removed from the experimental plots in late April each year, and then, the collected litter from the three plant species (clipped into fragments approximately 1cm in length) was distributed evenly by hand. Subsamples of the litter from each plant species had previously been oven-dried at 65°C to a constant mass to allow calculation of the moisture correction factor and were then weighed so that the initial litter mass could be estimated.

### Plant Community Surveys

Plant above-ground biomass was estimated by clipping plant material above the soil surface in randomly selected 0.5×0.5m quadrats within in each plot. Samples were collected from mid-June to August each year. All plant samples were classified according to their functional group (grasses, sedges, forbs, or legumes). Above-ground plant samples were oven-dried at 65°C to a constant weight and were then weighed to calculate the plant above-ground biomass of the different functional groups and the plant community. At the time when above-ground biomass samples were collected, plant height and canopy cover were measured in a permanent quadrat (0.5×0.5m) that had been established in each plot. Plant cover was estimated as the percentage of the surface area covered by all plant species present in the quadrat. Plant species richness and below-ground biomass were measured in each plot in mid-August (the peak of the growing season, with maximum species richness). Species richness was recorded as the total number of species detected in the quadrats. At the same time, soil samples were collected in each plot within the 0–10cm soil layer, the moisture content, total carbon, and total nitrogen were measured.

### Assessment of Plant Functional Group Evenness and Composition, and Community Biomass Stability

The plant functional group evenness index was calculated as: *E* = $\left(-\overset{S}{\underset{i=1}{\sum }}P_{i}\times \log P_{i}\right)/\ln S$, where *P_i_* is the relative above-ground biomass of plant functional group *i* in the community and *S* the number of plant functional groups (Andruschkewitsch et al., 2014). The proportion of above-ground biomass for each of the functional groups was used to calculate the changes in plant functional composition that had resulted from the litter treatments. These changes were expressed as a percentage, namely, (*B_i_*/*B_s_*)×100, where *B_i_* is the above-ground biomass of functional group *i* and *B_s_* is the community above-ground biomass of all species present in the quadrat. Legumes accounted for only 3.2% of the plant community biomass and were therefore grouped with forbs. As in previous studies, the plant community biomass stability was quantified as the ratio of the mean community above-ground biomass to its temporal standard deviation in each plot (Hautier et al., 2014).

### Assessment of Ecosystem Multifunctionality

Ten key ecosystem functions were selected, including both plant community functions (above- and below-ground biomass, species richness, functional group evenness, cover, and community biomass stability) and soil nutrient resources (moisture content, total carbon, total nitrogen, and carbon/nitrogen ratio), which together reflect ecosystem multifunctionality. The ecosystem multifunctionality was calculated using an averaging approach as described by Maestre et al. (2012b). We standardized each function by Z-score transformation observed in each plot and took the mean value across all functions in order to evaluate ecosystem multifunctionality at the plot level. Then, the average ecosystem multifunctionality values inverted around the 0 mean were converted to positive values by adding a constant (+1) to all values for further analysis. By doing this, the general difference among plant litter treatments in overall ecosystem functioning could be more easily assessed.

### Statistical Analysis

Key ecosystem functions and plant functional composition were analyzed with a repeated-measurements model using species, mass, date and interaction as fixed effects, and unit nested within blocks as random effects. The effects of litter manipulation on ecosystem multifunctionality were assessed with a linear mixed-effects model using litter species and litter mass as fixed effects, and plot as random effects. These analyses were performed using the “predictmeans” and “lme 4” packages in R 4.1.0 (R Core Team, 2021). In addition, we used piecewise structural equation modeling (SEM) to investigate the direct and indirect effects of plant community functional characteristics and litter manipulation on ecosystem multifunctionality. The model assumed that litter mass, litter species, and their interaction alter species richness, plant functional group evenness, and plant cover *via* changes in plant functional composition (extracting the first component scores from the principal component analysis conducted for proportion of above-ground biomass of three functional groups) and that they ultimately influence ecosystem multifunctionality. This was based on an *a priori* conceptual model of hypothetical relationships that include all of the different cascading pathways and all possible pathways (Supplementary Figure 1). We simplified the initial model by sequentially eliminating the non-significant pathways until the final optimal model was obtained. And the direct, indirect, and total effects of predictor variables on response variable in the conceptual model were calculated, respectively (Ali et al., 2019). Data were standardized by z transformation and used in the analysis, incorporating random effects in the plot. The d-separation tests, Fisher’s *C* statistic, and Akaike information criteria (AIC) were used to assess the goodness-of-fit of the model (Shipley, 2013). SEM analyses were performed using the “piecewiseSEM” package (Lefcheck, 2016) in R version 4.1.0 (R Core Team, 2021).

## Results

### Ecosystem Multifunctionality

**Figure 1:**
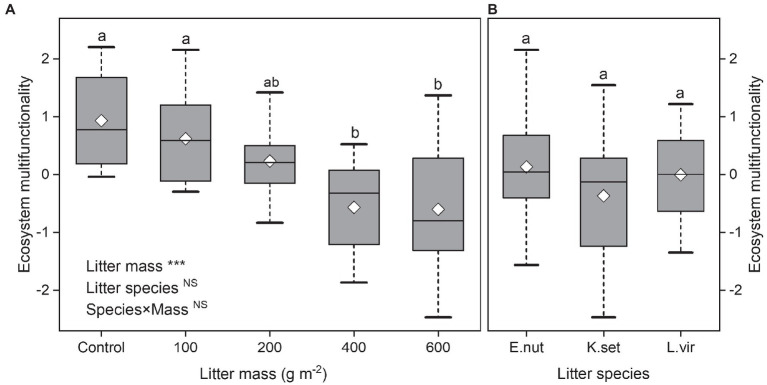
Effects of litter mass **(A)** and species **(B)** on ecosystem multifunctionality. Lowercase letters indicate significant differences at *p*<0.05 (^***^p<0.001; NS, not significant). Box plots show midline, mean; box edges, first quartile and third quartile; and whiskers, minimum and maximum. *E. nut*, *E. nutans*; *K. set*, *K. setchwanensis*; *L. vir*, *L. virgaurea*.

Ecosystem multifunctionality decreased significantly with increasing litter mass (bivariate regressions: *R*^2^=0.29, *p*<0.001). Litter species or interaction effect (species×mass) had no significant effect on ecosystem multifunctionality (*p*>0.05; Figures 1A,B). The ecosystem multifunctionality for 400 and 600gm^−2^ litter treatments was significantly lower than that for the control and 100gm^−2^ treatments.

### Plant Above-Ground Biomass and Cover

**Figure 2:**
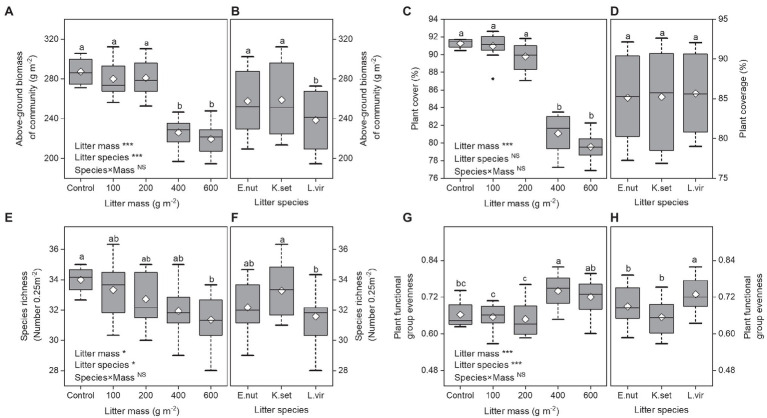
Effects of litter mass and species on plant communities. Above-ground biomass of community **(A,B)**, plant cover **(C,D)**, species richness **(E,F)**, and plant functional group evenness **(G,H)**. Lowercase letters indicate significant differences at *p*<0.05 (^*^p<0.05 and ^***^p<0.001; NS, not significant). Box plots show midline, mean; box edges, first quartile and third quartile; and whiskers, minimum and maximum. *E. nut*, *E. nutans*; *K. set*, *K. setchwanensis*; *L. vir*, *L. virgaurea*.

Over the study period, above-ground biomass of community and plant cover decreased linearly with increasing litter mass (Figures 2A,C). Litter species treatment had a significant effect on above-ground biomass of community (Figure 2B), whereas plant cover showed a non-significant response to litter species treatment (Figure 2D). The above-ground biomass of community and plant cover was significantly lower for 400 and 600gm^−2^ treatments compared with the other treatments (*p*<0.001). The plant community above-ground biomass was significantly higher for *E. nutans* and *K. setchwanensis* litter treatments than for the *L. virgaurea* litter treatment (*p*<0.001).

### Plant Diversity

Litter manipulation had marked effects on both species richness and plant functional group evenness, but the interaction effect (species×mass) was not statistically significant (Figures 2E–H). Species richness was significantly lower for the 600gm^−2^ litter treatment compared with the control (*p*<0.05) and was significantly higher for the *K. setchwanensis* litter treatment than for the *L. virgaurea* litter treatment (*p*<0.05). There was greater plant functional group evenness for the 400 and 600gm^−2^ litter treatments and for the *L. virgaurea* litter treatment (*p*<0.001).

### Plant Functional Composition

**Figure 3:**
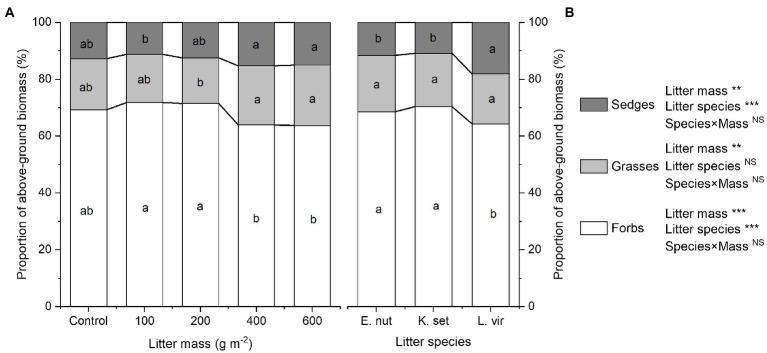
Effects of litter mass **(A)** and species **(B)** on plant functional composition. Summarized into proportion of above-ground biomass of forbs, grasses, and sedges. Lowercase letters indicate significant differences at *p*<0.05 (^**^p<0.01 and ^***^p<0.001; NS, not significant). *E. nut*, *E. nutans*; *K. set*, *K. setchwanensis*; *L. vir*, *L. virgaurea*.

Both litter mass and litter species had a significant impact on plant functional composition. The effect of the litter mass×species interaction on plant functional composition was not statistically significant (Figures 3A,B). For the 200gm^−2^ litter treatment, the proportion of forbs biomass was highest and the proportion of grasses biomass was lowest. The 400gm^−2^ and 600gm^−2^ litter treatments significantly decreased the proportion of forbs biomass compared with the 100 and 200gm^−2^ treatments (*p*<0.001). In contrast, the 400gm^−2^ and 600gm^−2^ treatments significantly increased the proportion of grasses biomass compared with the 200gm^−2^ treatment (*p*<0.01) and the proportion of sedges biomass compared with the 100gm^−2^ treatment (*p*<0.01). The proportion of forbs biomass was significantly lower for the *L. virgaurea* treatment than for the *K. setchwanensis* and *E. nutans* treatments (*p*<0.001), but the opposite result was obtained for the proportion of sedges biomass (*p*<0.001). Litter species had no significant effect on the proportion of grasses biomass (*p*>0.05).

### Drivers of the Litter Manipulation Effect on Ecosystem Multifunctionality

**Figure 4:**
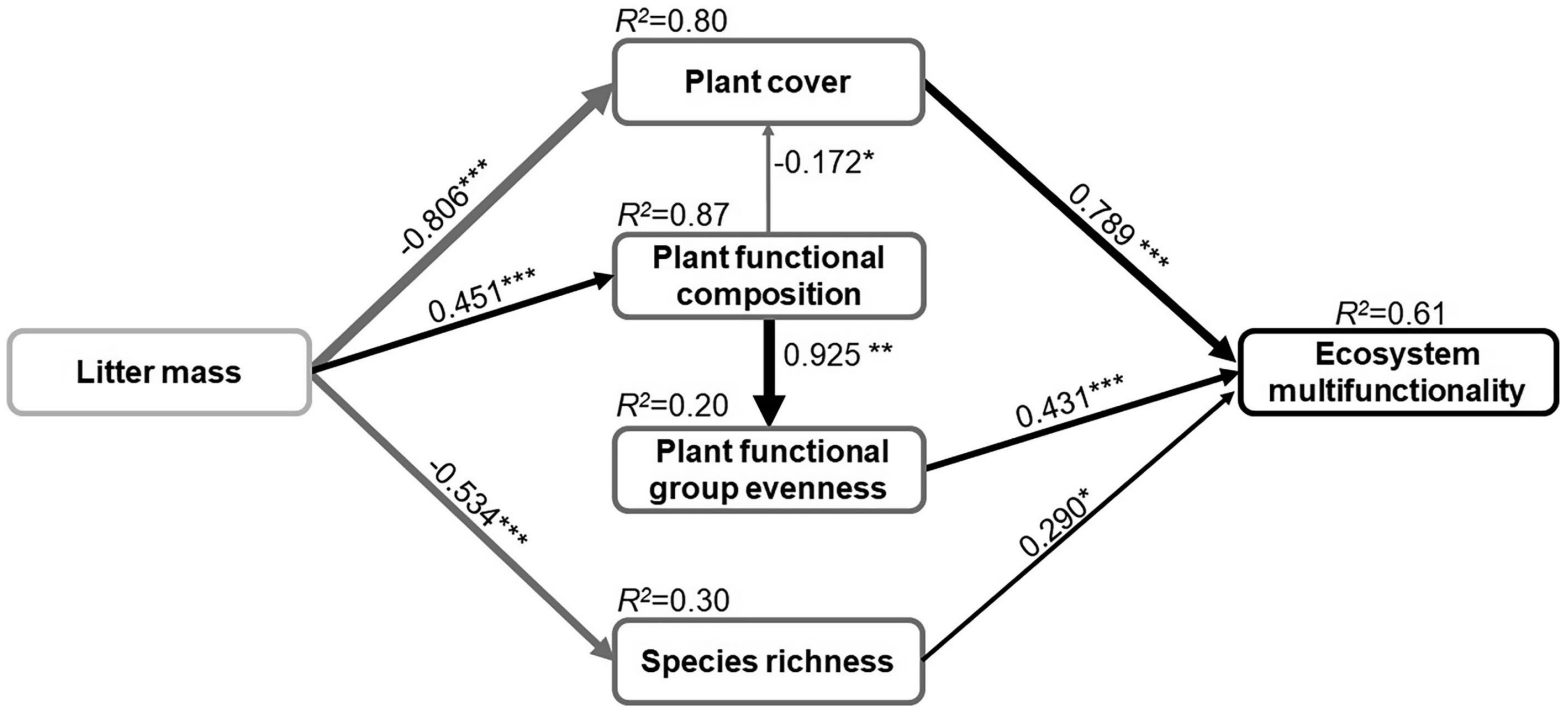
The path analysis result of piecewise structural equation model showing the direct and indirect causal relationships of litter manipulation and plant community properties effects on ecosystem multifunctionality. The black and gray solid arrows indicate that the positive and negative effect is significant, respectively (^*^p<0.05, ^**^p<0.01, and ^***^p<0.001). Arrow width and the associated number indicate the strength of the effect and the standard path coefficients, respectively. *R*^2^ values represent the proportion of variance explained for each dependent variable. Fisher’s *C*=14.82, value of *p*=0.39, and AIC=60.82.

In contrast to our hypothesis (Supplementary Figure 1), the final SEM predicted that only the litter mass has a significant impact on ecosystem multifunctionality (Figure 4; Supplementary Table 1). The SEM indicated that the litter mass response explained 64.4% of the variance in ecosystem multifunctionality. The positive and direct effects on ecosystem multifunctionality were mainly due to plant cover (path coefficient=0.79; *p*<0.001), and to a lesser extent to plant functional group evenness (path coefficient=0.43; *p*<0.001) and species richness (path coefficient=0.29; *p*<0.05; Figure 4). The SEM also indicated that the best predictor of ecosystem multifunctionality was plant cover and that it was directly influenced by litter mass (path coefficient=−0.81) and indirectly affected by plant functional composition (path coefficient=−0.17). Plant functional group evenness was indirectly affected by litter mass through plant functional composition (path coefficient=0.93), and species richness was directly affected by litter mass (path coefficient=−0.53).

## Discussion

Factors that contribute to ecosystem multifunctionality across biotic communities, such as ecosystem biodiversity and above-ground community composition, have been well studied in many terrestrial ecosystems (Jing et al., 2015; Xu et al., 2021). However, little is known about how plant diversity and most individual ecosystem functions under litter feedbacks directly or indirectly influence ecosystem multifunctionality, particularly when the litter mass of different plant litter species is quantitatively manipulated. The present study has shown that ecosystem multifunctionality decreases in response to increasing litter mass (Figure 1A). Moreover, plant cover and plant functional group evenness played a more important role than species richness in mediating the response of ecosystem multifunctionality to litter manipulation in an alpine meadow (Figure 4). However, the results also indicated potentially positive effects of low and medium amounts of litter accumulation (≤200gm^−2^) in maintaining ecosystem multifunctionality. Litter mass rather than litter species determined ecosystem multifunctionality under litter manipulation (Figure 1). These findings show that, in alpine grasslands, plant litter has a significant effect on most above-ground individual functions (Figure 2; Ma et al., 2021) and may be the primary driver of ecosystem multifunctionality.

### Plant Cover in the Response of Ecosystem Multifunctionality to Litter Manipulation

The plant cover was found in the present study to be the strongest indicator of litter effects on the ecosystem multifunctionality in an alpine meadow. Plant cover was particularly sensitive to litter manipulation and likely decreased through both the overspread of undecomposed litter and changes in functional group composition, thereby decreasing the ecosystem multifunctionality (Figure 4). Consistent with the findings of previous studies, plant cover positively influences the ecosystem multifunctionality (Soliveres et al., 2014). Likewise, Maestre and Escudero (2009) emphasized that plant cover was expected to affect ecosystem multifunctionality, as it largely modulated the effects of the soil variable. The physical and chemical effects of the higher litter mass inhibiting plant growth (Weltzin et al., 2005) or drastically increased the cover of functional groups (Wang et al., 2010), and thus indirectly reducing the vegetation cover. Limitation of light availability might also be the main reason for the reduction of plant cover under the litter manipulation (Deutsch et al., 2010).

### Plant Diversity in the Response of Ecosystem Multifunctionality to Litter Manipulation

Our study revealed that the litter mass manipulation significantly impacted the species richness and plant functional group evenness, thus impacting the ecosystem multifunctionality (Figure 4). The finding that has also been observed in several previous studies, where the species richness modulated by community attributes (e.g., spatial pattern; Maestre et al., 2012a) or climate (Jing et al., 2015) altered ecosystem multifunctionality, and the same direction as evenness effects (Lembrechts et al., 2018). In contrast, Soliveres et al. (2014) have reported no effect of species richness or evenness on ecosystem multifunctionality. Specifically, the SEM identified that the effect of plant functional group evenness on ecosystem multifunctionality was indirect, by altering the plant functional composition under litter mass treatment (Figure 4). The possible explanation is that the reduction in dominant functional groups creates conditions that are more favorable for the establishment of seedlings of rare functional groups (Liu et al., 2018), and thus increases plant functional group evenness. Consistent with this, Letts et al. (2015) reported that the increased functional evenness that results from the presence of larger amounts of litter is most probably attributable to an increase in nutrient availability. In the present study, higher litter mass also had a negative effect on species richness (Figure 2E), where increases in litter quantity have directly influenced species recruitment as a result of the effects of light limitation (Mariotte et al., 2016) or poor soil aeration (Cheng et al., 2011).

### Plant Functional Composition in the Response of Ecosystem Multifunctionality to Litter Manipulation

The SEM also identified the plant functional composition was key indirect driver of ecosystem multifunctionality, which can be attributed to the plant diversity and plant cover that are determined by the transformation of plant functional groups (Figure 4). In particular, the substantial reductions in the proportion of above-ground biomass of forbs may be the main reason impacting ecosystem multifunctionality, and to a lesser extent by an increase in that of grasses and sedges. This can be attributed to the approximately more than 60% of above-ground biomass of forbs in the experimental community (Figure 3A). Other studies have also previously observed that the plant functional composition contribute more to the ecosystem multifunctionality, probably because the more proportion of grass and sedge is more likely to improve soil nutrient (Xu et al., 2021) and below-ground biomass (Song et al., 2020b).

In our study, the dynamics of plant functional composition may be related to its ability to withstand various environmental perturbations. Plant litter may promote plant strategies that favor resource allocation for particular modules and may thereby drive dynamics that optimize vegetation composition (Kaproth et al., 2013). For example, Liu et al. (2018) found that shallow-rooted forbs were more sensitive to experimental manipulations than were deep-rooted grasses. In addition, the amount of litter can significantly affect photosynthetically active radiation (Zhang et al., 2019) and allelochemical content (Loydi et al., 2015), and thus increase the dominance of grass and sedge species. The observed reductions in the proportion of above-ground biomass of grasses were probably due to the vertical structure of grasses and their rhizome propagation characteristics in the plant community (Mao et al., 2015). Our findings are consistent with those of other studies that have been conducted in annual grassland (Amatangelo et al., 2008).

In particular, we still found that litter species of *L. virgaurea* had a stronger effect on above-ground biomass, plant diversity, and plant functional composition (Figures 2B,F,H, 3B). It is probably because litter species of *L. virgaurea* decomposes more quickly, affecting nutrient dynamics and driving effects that are attributable to phytotoxic compounds (Liang, 2013; Saito et al., 2015).

### Implications for Ecosystem Management and Sustainability Based on Multifunctionality

Overall, our results have important implications for maintaining ecosystem multifunctionality in alpine meadows located on the QTP. Although grazing exclusion has been proposed and implemented for the mitigation of degraded grasslands, not all of the degradation of ecosystem functions has been mitigated (Zou et al., 2016; Sun et al., 2020). Our findings support the hypothesis that high levels of litter accumulation have a negative impact on ecosystem multifunctionality. In addition, the present study found that low and medium levels of litter accumulation (≤200gm^−2^) were potentially beneficial for maintaining optimal ecosystem multifunctionality. Therefore, moderate grazing disturbance (e.g., low grazing intensity) is needed in order to achieve optimal litter accumulation levels and maximize the sustainability of ecosystem multifunctionality (Ren et al., 2018; Zhang et al., 2021). The results of our study increase our understanding of how litter manipulation can regulate ecosystem multifunctionality and will help to raise awareness of how ecosystem functions respond to such litter accumulation dynamics. These research findings therefore have important implications for achieving sustainable ecosystem multifunctionality in relation to litter accumulation dynamics and heterogeneity in the alpine meadows of the QTP region. They also suggest that ecosystem multifunctionality will be reduced under unsuitable grassland management regimes. Therefore, it is vitally important to optimize grassland management so as to minimize the negative impact of litter on the sustainable development of ecosystem multifunctionality.

## Conclusion

This study has demonstrated that litter mass manipulation involving the application of high levels of litter significantly reduces ecosystem multifunctionality in an alpine meadow on the QTP. Ecosystem multifunctionality reaches a critical threshold after treatment with a litter mass of 200gm^−2^. Our findings highlight the potential role of plant diversity (species richness and plant functional group evenness) and plant cover in maintaining ecosystem multifunctionality under litter feedbacks, and the likely dependence on the dynamics of plant functional composition. Litter-induced feedback has fundamental implications for optimizing ecosystem multifunctionality while simultaneously maintaining biodiversity and ecological functions. In addition, it is suggested that appropriate grassland management measures need to regulate the accumulation of plant litter, which has a better driving and maintenance effect on the regulation of ecosystem multifunctionality in alpine meadows.

## Data Availability Statement

The original contributions presented in the study are included in the article/Supplementary Material, further inquiries can be directed to the corresponding author.

## Author Contributions

FH conceived the ideas and designed the experiment. ZM and JW conducted the field experiment and collected the data. FH, ZM, and LL performed all the statistical analyses and modeling. FH and ZM led the writing of the manuscript. All authors revised the manuscript and contributed critically to the drafts and gave final approval for publication.

## Funding

This research was supported by the Second Tibetan Plateau Scientific Expedition: Grassland Ecosystem and Ecological Animal Husbandry (2019QZKK0302), the National Natural Science Foundation of China (31672472), the Program for Innovative Research Team of Ministry of Education (IRT17R50), the Technological Support for Grassland Ecological Management and Restoration and the Pastoral Livestock Industry in Gansu Province, China (GARS-08), and “Lanzhou City’s Scientific Research Funding Subsidy to Lanzhou University”.

## Conflict of Interest

The authors declare that the research was conducted in the absence of any commercial or financial relationships that could be construed as a potential conflict of interest.

## Publisher’s Note

All claims expressed in this article are solely those of the authors and do not necessarily represent those of their affiliated organizations, or those of the publisher, the editors and the reviewers. Any product that may be evaluated in this article, or claim that may be made by its manufacturer, is not guaranteed or endorsed by the publisher.
