# Supplementary material for: Litter-Induced Reduction in Ecosystem Multifunctionality Is Mediated by Plant Diversity and Cover in an Alpine Meadow
Source: Front Plant Sci. 2021 Nov 25;12:773804. doi: 10.3389/fpls.2021.773804 (PMC8656275; doi:10.3389/fpls.2021.773804)
Supplement: Supplementary file 1 [file Data_Sheet_1.docx]

**Supplementary Material**

**Litter****-induced reduction in** **ecosystem** **multifunctionality is mediated by plant diversity and cover in an alpine meadow**

Zhouwen Ma ^1^, Jing Wu ^1^, Lan Li ^1^, Qingping Zhou ^2^, Fujiang Hou ^1^*

^1^ State Key Laboratory of Grassland Agro-ecosystems, Key Laboratory of Grassland Livestock Industry Innovation, Ministry of Agriculture and Rural Affairs, College of Pastoral Agriculture Science and Technology, Lanzhou University, Lanzhou 730020, China, ^2^ Institute of Qinghai-Tibet Plateau, Southwest Minzu University, Chengdu, 610041, China

***Corresponding author**

Fujiang Hou, College of Pastoral Agriculture Science and Technology, Lanzhou University. E-mail: [cyhoufj@lzu.edu.cn](mailto:cyhoufj@lzu.edu.cn)


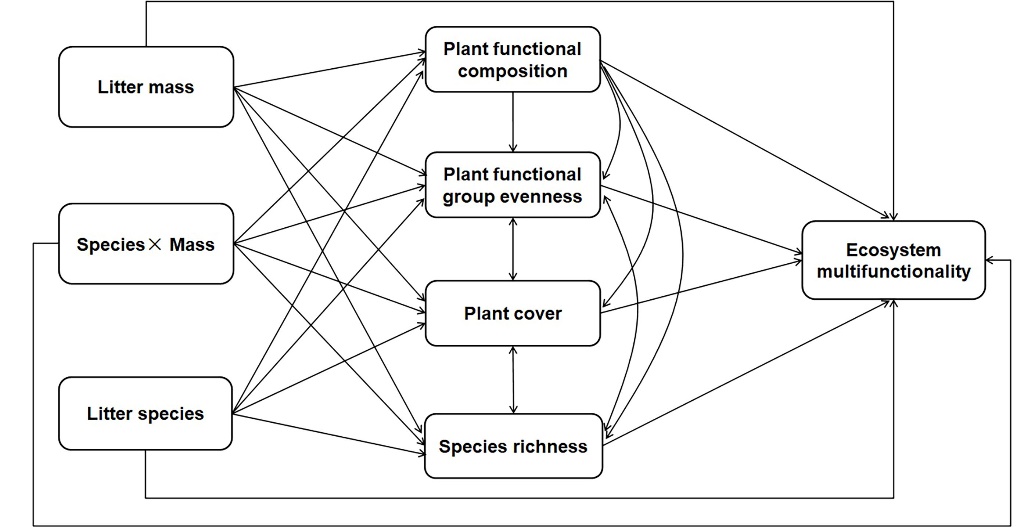


**Supplementary Figure 1.** Hypothetical causal model for structural equation modelling (SEM) exploring the effects of litter mass, litter species, the interaction of litter mass and litter species, plant functional composition, plant cover, species richness, plant functional group evenness on ecosystem multifunctionality in the litter manipulation experiment. Black arrows are hypothesized paths.

**Supplementary Table 1.** Summary of the predictive effects of structural equation modeling (SEM) depicting litter mass, plant functional composition, plant functional group evenness, species richness and plant cover on ecosystem multifunctionality. Shown are the direct and indirect effect coefficient.

| Response variable | Predictor variable | Effect | Coefficient |
| --- | --- | --- | --- |
| Ecosystem multifunctionality | Litter mass | Indirect | -0.471 |
| Ecosystem multifunctionality | Plant functional composition | Indirect | 0.266 |
| Ecosystem multifunctionality | Plant functional group evenness | Direct | 0.431 |
| Ecosystem multifunctionality | Species richness | Direct | 0.290 |
| Ecosystem multifunctionality | Plant cover | Direct | 0.789 |
| Plant cover | Litter mass | Direct | -0.806 |
| Plant cover | Plant functional composition | Indirect | -0.172 |
| Plant cover | Litter mass | Indirect | -0.078 |
| Plant functional composition | Litter mass | Direct | 0.451 |
| Plant functional group evenness | Plant functional composition | Direct | 0.931 |
| Plant functional group evenness | Litter mass | Indirect | 0.420 |
| Species richness | Litter mass | Direct | -0.534 |
